# Predictors of quality of life of older persons in rural Uganda:  A cross sectional study

**DOI:** 10.12688/aasopenres.12874.2

**Published:** 2018-11-09

**Authors:** Fred Maniragaba, Betty Kwagala, Emmanuel Bizimungu, Stephen Ojiambo Wandera, James Ntozi

**Affiliations:** 1 Department of Population Studies, Makerere University, Kampala, 7062, Uganda; 2 International Food Policy Research Institute, Kampala, Uganda

**Keywords:** Ageing, older persons, elderly persons, later life, quality of life

## Abstract

**Background:** Little is known about the quality of life of older persons (OPs) in Uganda in particular, and Africa in general. This study examined factors associated with quality of life of older persons in rural Uganda.

**Method:** We performed a cross-sectional survey of 912 older persons from the four regions of Uganda. Data were analyzed at univariate, bivariate and multivariate level where ordinal logistic regression was applied.

**Results:** Older persons in northern (OR=0.39; CI=0.224-0.711) and western (OR=0.33; CI=0.185-0.594) regions had poor quality of life relative to those in central region. Those who were HIV positive had poor quality of life (OR=0.45; CI=0.220-0.928) compared to those who were HIV negative. In contrast, living in permanent houses predicted good quality of life (OR=2.04; CI=1.391-3.002). Older persons whose household assets were controlled by their spouses were associated with good quality of life (OR=2.06;CI=1.032-4.107) relative to those whose assets were controlled by their children.

**Conclusion:** Interventions mitigating the HIV and AIDS related Quality of life should target older persons. The government of Uganda should consider improving housing conditions for older persons in rural areas.

## Introduction

The world is experiencing remarkable and irreversible demographic changes as many people are living longer than ever before ^1^. Population ageing is a dynamic process that involves physical, psychological and social changes ^2^. It is expected to advance faster in the developing world including sub-Saharan Africa (SSA) ^3^. Older persons are defined as persons aged 60 years and older ^3^. Although the World Health Organization (WHO) uses 50 years and above to define older persons for Africa ^4^, this study used United Nation’s definition of age 60 years and older, since Uganda’s life expectancy is 63 years ^5^. Globally, one in every nine persons is an older person. This ratio is expected to reduce to one person in every five people by 2050 ^2, 6^. 

Uganda’s population is largely characterized by young people. Nonetheless, the country’s older population has more than doubled from 686,000 in 1991 to 1,433,596 in 2014 (UBOS 2016. This is attributed to the reduction in adult mortality ^5, 7^. This is an unprecedented phenomenon that requires strategic planning.

Just like in other developing countries where population ageing is taking place, the older people in Uganda experience a diversity of challenges ^8^. These range from loneliness, poor housing, and financial challenges to poor health particularly non communicable diseases ^9^. The situation is compounded by poverty and limited access to health services which ultimately affect their quality of life ^2, 8, 10, 11^.

Many older persons lack social security which makes it difficult for them to meet their basic needs ^10, 12^. Only a small proportion (7.1%) have access to pension ^7^. Close to a half (45%) of the older persons in Uganda have physical disabilities ^10^ which limit their access to health services. These challenges are worse in rural areas where older persons are particularly vulnerable owing to financial resource limitations, long distances to health facilities, discrimination at health facilities and in matters of social engagements ^2, 11^. 

The Government of Uganda has put in place policies and programmes such as social assistance grant for empowerment (SAGE) and decentralization of retirement benefits with the aim of improving OP’s quality of life ^7^. The SAGE is being piloted in 40 out of 121 districts. New hospitals are also being constructed while old ones are undergoing renovation to ease accessibility to the health services ^10^. However, more remains to be done. For instance, since most people are employed in the informal sector, economic empowerment through social security systems does not cater for the well-being of the majority older persons ^10^.

Research on older persons in Uganda has focused on areas such as vulnerability of older adults ^13^, chronic poverty among older persons ^14^, nutritional status and functional ability of the older persons ^15^ and understanding the vulnerability of older adults: extent of and breaches in support systems in Uganda ^16^. Although these studies address important aspects of population ageing, comprehensive evidence regarding quality of life of older persons remains scanty in Uganda. None of these studies focused on the predictors of quality of life of older persons in rural Uganda. Some of these studies such as that done by Kikafunda and Lukwago ^15^ utilized data from a limited geographical scope while others ^13, 14^ used secondary data collected some years back. Therefore, to bridge the highlighted knowledge gap, our study builds on the existing body of literature using the most recent dataset collected from a wide geographical scope to evaluate the factors associated with perceived quality of life of older persons in rural Uganda.

## Methods

This paper is based on a cross sectional study on predictors of quality of life of older persons in rural Uganda. The data were collected from February to March, 2017. The study included both men and women aged 60 years and above. It excluded those who were ill or lacked capacity to provide rational voluntary consent. We did not interview OP’s who were ill. The challenge with interviewing such ill OPs is that some are too weak to sustain the interview as they need to rest. We note, however that this exclusion can bias the outcome of the study. A sample of 912 respondents was calculated basing on survey sampling ^17^. Multi-stage stratified cluster sampling was used to select respondents from Uganda’s geographical regions of central, eastern, northern and western which constituted the strata. The districts, sub counties, villages and households were randomly selected ( Table 1). In each region, one district was selected within which three sub-counties were also randomly selected. Four villages were selected per sub-county, providing a total of 12 villages per district and hence 48 villages across the country. Simple random sampling was used at each stage of selection.

**Table 1.** Selected study areas.

Region	Selected district	Sub-county	Selected villages
**Central**	Kayunga	Busana	Bugadu
			Kawuku
			Busaana
			Namirembe
		Kitimbwa	Wabwoko
			Kitimbwa
			Kyelima
			Nongo
		.Nazigo	Kiteredde
			Bukamba
			Buguvu
			Katikanyonyi
**Eastern**	Bugiri	Iwemba	Waliko
			Nambobi
			Kazimba
			Nabyunu
		Kapyang	Bugubo
			Muyemu
			Bukaye
			Bugodo
		Nabukalu	Bulalo
			Bukazito
			Kakoge
			Bulanga
**Northern**	Otuke	Orum	Ochokoimaki
			Amunga
			Ogwar
			Camukoki
		Ogor	Olet
			Awei
			Alei
			Ojany
		Adwari	Abworoyere
			Ecamoweno
			Okwongo west
			Onger
**Western**	Kabale	Bubare	Nyamiyaga
			Muchahi
			Kitumbezi
			Kashaki
		Nyamweru	Bigungiro
			Keru
			Rwakaruma
			Kagoye
		Hamurwa	Hakaburara
			Rwamuguru
			Muruhita
			Kamuterere

We obtained verbal consent from the village leaders who helped to identify and list households with at least one member aged 60 years and above. We used the lists to randomly select the planned sample of ten households from each village. Thus, a total of 480 households were drawn for interviews.

This study was conceptualized basing on World Health Organization Quality of Life (WHOQOL-BREF) and World Health Organization Quality of Life (WHOQOL-OLD) instruments ^18, 19^. The WHOQOL-BREF consists of 26 questions, 24 of which are divided into four domains: physical health, mental health, social relationships, and environment. This study adapted with modifications the questions concerning physical health dimension of quality of life. The items under this dimension include activities of daily living, mobility status, energy and fatigue; body pain and sleep ^20^. The WHOQOL-OLD ^18^ comprises 24 items (rated on a 5-point Likert-type scale), divided into 6 facets namely; sensory abilities; autonomy; past, present, and future activities; social participation; death and dying; and intimacy. We adapted with modification questions about social participation such as; sufficient number of activities to perform each day, satisfaction with one’s use of time, social activities and participation in community activities. We also considered intimacy with items such as; feeling a sense of companionship in life, experiencing love, and having opportunities to love and be loved. Full questionnaire used for this study is available as Supplementary File 1.

Ethical clearance for the study was granted by National HIV/AIDS research committee (NARC, Ref: ARC 190), Uganda National Council for Science and Technology (UNCST, Ref: SS 4167) and Office of the President of the Republic of Uganda (Ref: ADM 194/212/01). While collecting data, verbal informed consent was obtained from all respondents prior to the interviews and they were assured of utmost confidentiality. The consent was verbal because the respondents were not comfortable with writing due to old age. All information were given in local languages (Rukiga, Langi, Luganda and Lusoga).

The dependent variable is quality of life formed from the three latent constructs (social participation, physical health and intimacy). For each latent construct, three categorical responses were generated (good, fair and poor). These responses were summed up for each older person to generate corresponding categorical responses for quality of life. Physical health covered 5 areas namely, activities of daily living, mobility difficulties, sleep, body pain and fatigue; social participation covered 3 areas such as satisfied with usage of time, social activities, enough physical activity to do daily and participate in family and community; and intimacy covered 3 areas such sense of companionship, opportunity to be loved and sexual activity ^18^.

Data were analyzed at univariate, bivariate and multivariate levels using STATA software (version 15). Due to the complex nature of the sampling design, sampling weights were calculated to correct imperfections resulting from selection of units with unequal probabilities and ensure representativeness of the results. At univariate level, a basic description of the respondents is done. At bivariate level, chi-square tests were performed to examine the association between the dependent and independent variables. At multivariate level, we employed factor analysis (FA), which is part of multivariate analysis technique. Therefore, using different indicators including physical health, intimacy and social participation which measure slightly different aspects of quality of life would not be very practicable in analyzing the factors that determine the quality of life. However, high correlation among these indicators could help to produce a lower number of latent variables that fit common patterns in the data. The (FA) was employed to identify variables that factor well together and with notable loading magnitude in absolute terms. Based on the number of factors extracted, an index for the identified factors was calculated through linear combination between observed and factor loadings. Bartlett test of sphericity and the Kaiser-Meyer-Olkin (KMO) criterion were performed to verify whether indicators of each category shared a common core. The Bartlett test was used to estimate the probability that the correlation matrix is zero, implying that all the variables are uncorrelated, while the KMO was used to indicate the extent to which variables had common feature to warrant factor analysis. In the Supplementary Table 1, KMO scores above 0.4 (threshold scores) were acceptable; scores above 0.9 were exceptional ^21^. In this study, the analysis yielded a KMO value of 0.814, while the Bartlett test yielded $\chi_{\text{253}}^{2}$ = 7057.335 ( *p=0.000*), signifying the data’s adequacy for factor analysis. The number of factors to retain was determined based on the eigenvalue criteria and the scree plot ( Supplementary Figure 1).

The outcome variable (QOL) was generated through an index generated from an aggregation of factor analysis scores for the three latent constructs: physical health, social participation and intimacy. Using STATA’s tertile grouping technique, three categorical responses were automatically generated with the first group indicating higher values, the second group indicated medium values while the smallest group was a representation of the lowest values. The first group was labelled ‘good’ quality of life, the second group was labelled ‘fair’ quality of life while the third (last) group was labelled ‘poor’ quality of life. The ordered logistic regression model was used for the final analysis owing to the rating of people’s quality of life whereby, some individuals are expected to be better off or worse off than others. The strengths of the relationships were reported as odds ratios. The p-value was used to determine whether the relationship was significant or not depending on the level of significance which was fixed at 0.05.

## Results

### Descriptive characteristics of older persons

Results in Table 2 show that over half (51%) of the respondents were age 60–69 years and majority (57%) were females. Over half (51%) were married. Regional representation ranged from 21% for Northern to 27% for the central region. The large majority (54%) had no formal education, primary (40%) and as expected, tertiary came last at 3%. Over one-third (38%) of the respondents were either affiliated to the Anglican or Catholic faith.

**Table 2.** Percent distribution of respondents by selected socio-demographic characteristics.

Variable	Number	Percent (%)
Age		
**60 – 69**	462	50.7
**70 – 79**	290	31.8
**80+**	160	17.5
Sex		
**Male**	395	43.3
**Female**	517	56.7
Marital status		
**Married**	461	50.6
**Widowed**	361	39.5
**Divorced/Separated**	90	9.9
Region		
**Central**	248	27.2
**East**	237	25.9
**North**	190	20.8
**West**	237	25.9
Education level		
**No education**	495	54.3
**Primary**	363	39.8
**Secondary**	31	3.4
**Tertiary**	23	2.5
Religion		
**Catholic**	342	37.5
**Anglican**	350	38.4
**Muslim**	118	12.9
**Pentecostal**	71	7.8
**SDA**	20	2.2
**Other**	11	1.2

### Association between older persons’ quality of life and selected demographic and socio-economic factors

Table 3 presents chi-square test results for the association between quality of life and selected demographic and socio-economic factors. Age of the respondents, sex, marital status, ownership of a radio set, control over household assets, living arrangement, HIV sero status, healthcare taker during sickness and fuel type were not significantly associated with quality of life. Western region had the largest proportion of respondents with poor quality of life (52%; p<0.001), tertiary education (44%, p=0.006), residing in semi-permanent houses (43%; p=0.036), distance of 0–05Km to the nearest health center (52%; <0.001), using protected well/spring (51%; p<0.001). Furthermore, quality of life was poorer among older persons who were using other sources of energy for lighting like torch and candle (48%; p=<0.001) and those who were using covered pit latrine (39%; p=0.012).

**Table 3.** Association between older persons’ quality of life and selected demographic and socio-economic factors.

Variable	Observations (n = 912)	Perceived quality of life			*χ* ^2^	p-value
		Poor	Fair	Good		
**Age of the respondent**						
**60 – 69**	462	37.9	49.4	12.7		
**70 – 79**	290	36.9	48.6	14.5	2.3	0.688
**80 +**	160	31.9	53.8	14.4		
**Sex**						
**Male**	395	36.7	49.4	13.9		
**Female**	517	36.3	50.3	13.4	0.1	0.951
**Region**						
**Central**	248	29.4	54.0	16.6		
**East**	237	24.1	58.2	17.7	53.8	**<0.001**
**North**	190	42.1	43.7	14.2		
**West**	237	51.9	42.2	5.9		
**Education level of the respondent**						
**No education**	495	31.3	52.3	16.4		
**Primary**	363	43.3	46.6	10.1		
**Secondary**	31	35.4	58.1	6.5	18.1	**0.006**
**Tertiary**	23	43.5	39.1	17.4		
**Marital status of the respondent**						
**Married**	461	38.8	48.8	12.4		
**Widowed**	361	34.6	50.7	14.7	2.8	0.589
**Divorced**	90	32.2	52.2	15.6		
**Type of house respondent resides in**						
**Permanent**	223	34.5	53.8	11.7		
**Semi-permanent**	320	42.8	44.1	13.1	10.3	**0.036**
**Temporary**	369	32.3	52.6	15.1		
**Household’s radio set ownership**						
**Yes**	479	36.5	50.3	13.2	0.2	0.913
**No**	433	36.5	49.4	14.1		
**Control over household assets**						
**Self**	693	36.5	49.5	14.0		
**Spouse**	132	36.4	48.5	15.1		
**Children**	87	36.7	55.2	8.1	2.85	0.583
**Living arrangement**						
**Alone**	80	33.8	46.2	20.0		
**Spouse only**	22	54.5	40.9	4.6	11.2	0.083
**Spouse & children**	133	44.3	45.1	10.5		
**Children & grandchildren**	677	34.7	51.6	13.7		
**Whether respondent has HIV/AIDS**						
**Yes**	28	46.4	50.0	3.6		
**No**	854	37.0	49.2	13.8	2.8	0.252
**Distance to the nearest health center**						
**0 – 0.5 Km**	170	52.3	40.0	7.7		
**1 –2 Km**	426	36.6	50.0	13.4	30.3	**<0.001**
**> 2 Km**	309	27.8	55.0	17.2		
**Healthcare taker during sickness**						
**Spouse**	308	40.2	48.1	11.7		
**Children**	377	36.6	48.0	15.4	11.8	0.066
**Grandchildren**	91	24.3	64.8	10.9		
**Others**	136	36.0	49.3	14.7		
**Water source for the household**						
**Borehole/Piped water**	585	34.5	52.0	13.5		
**Protected well/spring**	183	50.8	38.8	10.4	24.4	**<0.001**
**Unprotected source**	144	26.4	55.6	18.0		
**Fuel type for the household**						
**Firewood**	871	35.9	50.1	14.0		
**Charcoal**	41	48.8	46.3	4.9	4.3	0.118
**Source of energy for the household**						
**Electricity**	121	47.9	43.8	8.3		
**Tadooba**	590	30.2	53.4	16.4	32.5	**<0.001**
**Others**	201	48.2	43.3	8.5		
**Type of Toilet for the household**						
**Covered pit latrine**	699	39.2	47.6	13.2		
**Uncovered pit latrine**	165	27.9	59.4	12.7	12.9	**0.012**
**Bush**	48	27.1	50.0	22.9		

*χ* ^2^=Pearson Chi square test

### Multivariate results

Table 4 presents the results of the ordered logistic regression model of factors influencing quality of life of older persons. Controlling for other variables, region of residence, type pf house, ownership of a radio, control over household assets and HIV sero status predicted quality of life. Region is a predictor of older persons’ quality of life. Compared to older persons from central region, the likelihood of good quality of life decreased among those from northern (OR=0.39; CI=0.224-0.711) and western regions (OR=0.33; CI=0.185-0.594). We also find that the type of house older person resides in as well as ownership of a radio as important predictors of their quality of life. Compared to older persons staying in semi-permanent houses, the odds of good quality of life increased (OR=2.04; CI=1.391-3.002) among those residing in permanent houses. The odds further increased (OR=1.73; CI=1.246-2.390) among those who possessed a radio compared to those who did not. Older persons whose household assets were controlled by their spouses (OR=2.06; CI=1.032-4.107) were more likely to have good quality of life relative to those whose assets were controlled by their children. Being HIV positive was associated with poor quality of life (OR=0.45; CI=0.220-0.928) among older persons relative to being HIV negative.

**Table 4.** Predictors of older persons’ quality of life.

Variable	Odds ratio	95% CI	*p-value*
**Age of the respondent (rc=60–69)**			
** 70 – 79**	1.073	[0.755-1.525]	0.695
** 80 – 89**	1.000	[0.636-1.573]	1.000
**Sex of the respondent (rc=female)**			
** Male**	1.259	[0.876-1.811]	0.213
**Region (rc=central)**			
** East**	1.025	[0.684-1.536]	0.906
** North**	0.399	[0.224-0.711]	**0.002**
** West**	0.332	[0.185-0.594]	**<0.001**
**Education level (rc=tertiary)**			
** No education**	0.819	[0.221-3.031]	0.765
** Primary**	0.538	[0.146-1.984]	0.352
** Secondary**	0.489	[0.111-2.165]	0.346
**Marital status (rc=married)**			
** Widowed**	1.382	[0.895-2.132]	0.144
** Divorced/separated**	1.356	[0.783-2.348]	0.277
**Type of house (rc= semi-permanent)**			
** permanent**	2.043	[1.391-3.002]	**<0.001**
** Temporary**	0.832	[0.558-1.241]	0.368
**Radio set ownership (rc=no)**			
** Yes**	1.726	[1.246-2.390]	**0.001**
**Control over household assets (rc=children)**			
** Self**	1.426	[0.857-2.374]	0.172
** Spouse**	2.059	[1.032-4.107]	**0.040**
**Living arrangement (rc=alone)**			
** Spouse only**	0.795	[0.238-2.655]	0.709
** Spouse & Children**	1.459	[0.697-3.056]	0.316
** Children & Grandchildren**	0.997	[0.565-1.763]	0.994
**Has HIV/AIDS (rc=no)**			
** Yes**	0.452	[0.220-0.928]	**0.031**
**Distance to the nearest health center (rc= >2km)**			
** 0–0.5Km**	0.644	[0.407-1.021]	0.061
** 1–2 Km**	0.987	[0.706-1.378]	0.937
**Water source (rc= borehole/piped water)**			
** Protected well/spring**	0.747	[0.442-1.263]	0.276
** Unprotected source**	1.558	[0.960-2.529]	0.073
**Fuel type (rc=firewood)**			
** Charcoal**	0.556	[0.263-1.176]	0.124
**Source of energy (rc=electricity)**			
** Tadooba**	1.180	[0.715-1.949]	0.517
** Others**	0.707	[0.386-1.296]	0.263
**Type of toilet facility (rc=covered pit** **latrine)**			
** Uncovered pit latrine**	0.846	[0.568-1.264]	0.417
** Bush**	1.049	[0.498-2.207]	0.900

***rc =reference category***

## Discussion

Age of the respondents, sex, education level, marital status of the OPs, living arrangement, distance to the nearest health facility, water source, fuel type, source of energy and type of toilet facility did not predict the quality of life of older persons in rural Uganda. Concerning region, the poor quality of life for older persons in northern Uganda could perhaps be linked to the protracted war that economically crippled the region. This finding is consistent with previous studies ^11, 22– 25^ which show that the quality of life of older persons is influenced by living in a safe, resource rich and desirable environment with. In western Uganda particularly Kigezi sub-region where data for this study were collected, poor quality of older persons is in consonance with studies such as Uganda National Household Survey ^26^ which indicates that the region suffers from a shortage of basic necessities such as inadequate food production.

The increase in odds of good quality of life for the older persons residing in permanent houses imply a better standard of living. Previous studies ^27– 32^ show that older persons whose households are of high economic status are associated with high quality of life.

Owning a radio set increased the odds of good quality of life. It could be noted that radios just like other household assets such as mobile phones and television not only disseminate health information, they also help to entertain OPs and keep them mentally pre-occupied and less lonely. Previous studies indicate that media apparatus entertain older persons and help to resolve loneliness and associated consequences like stress which is detrimental to the quality of life ^8^. Similarly, previous studies in Canada and Norway ^22^, Uganda ^11^ and other countries ^23– 25, 33, 34^ found that conducive environmental conditions characterized by easy access to information is good for healthy living of older persons.

Spousal control over household assets predicted good quality of life of older persons. It could be suggested that unlike children who could probably migrate to urban areas perhaps after selling off their family resources, the spouses of older persons could offer appropriate care which enhanced their partners’ quality of life. Our finding is in conformity with other studies in China ^35^ and other countries ^36^ which show that social support from their spouses stimulate OPs quality of life and improved longevity.

As expected, a positive HIV sero status was associated with poor quality of life. This finding is in conformity with previous studies which show that HIV/AIDS affects the health of the older persons and increases later life mortality ^37– 40^

### Limitations

Our study was intended to bridge the knowledge gap regarding the quality of life of older persons in Uganda. However, one major methodological limitation of this study is that the authors were unable to take anthropometric measurements, which could have enriched the results, due to limited funding. Furthermore, we did not interview the respondents who were ill. The exclusion of those who were ill could have potentially biased the results. The ill OPs were those reported to be in critical condition that would not enable them to participate in an interview.

## Conclusions and policy implications

Older persons’ quality of life was associated with the geographical setting, quality of shelter, resource control and health status. Interventions point to meeting basic needs of shelter, information and health needs of OPs. Policy intervention should also focus on deepening universal and equal access to information by older persons through subsidizing radio prices. This will encourage older persons to own the radios which would avail them with health information from health programmes and minimize loneliness through entrainment. Intervention in strengthening policies focusing on the older persons against HIV/AIDS pandemic should be fast-tracked through initiating adult safe sex education and improved access to Anti-Retroviral drugs especially for those who are already infected. The vulnerability of older persons in terms of access and control of resources calls for policy makers to strengthen economic strengthening strategies with a view of improving self-reliance of older persons. Furthermore, Uganda government’s intervention to mitigate regional disparities in quality of life of OPs should put special attention to northern and western regions where older persons’ quality of life is poor.

## Data availability

The data underlying this study is available from Open Science Framework: Dataset 1. Predictors of quality of life of older persons in rural Uganda: A cross sectional study https://doi.org/10.17605/osf.io/vfb4w ^41^

This dataset is available CC0 1.0 Public domain dedication.

## Supplementary material

Supplementary Table 1: Summary of rotated factor loadings against indicators (n = 912).

Supplementary Figure 1: Screeplot of eigenvalues that determined the retention of latent constructs.

Supplementary File 1: Questionnaire.
