# Supplementary material for: Predictors of quality of life of older persons in rural Uganda: A cross sectional study
Source: AAS Open Res. 2018 Nov 9;1:22. Originally published 2018 Jul 26. [Version 2] doi: 10.12688/aasopenres.12874.2 (PMC7118782; doi:10.12688/aasopenres.12874.2)
Supplement: Supplementary file 1 [file aasopenres-1-13995-s0000.tgz › c178b774-4e30-4609-91a8-b304a98d0495.docx]

**Supplementary Table 1: Summary of rotated factor loadings against indicators (n = 912)**

|  | Factor 1  Social participation | Factor 2  Physical health | Factor 3  Intimacy |
| --- | --- | --- | --- |
| **Social participation** |  |  |  |
| Participates in religious functions | **0.8488** | 0.0214 | -0.0332 |
| Participates in elderly clubs | 0.2892 | 0.1520 | -0.0230 |
| Participates in local ceremonies | **0.6531** | -0.0679 | 0.0602 |
| Participates in visiting friends | **0.7777** | 0.0528 | 0.0396 |
| Participates in charity walks | 0.1845 | 0.1821 | 0.0465 |
| Participates in social gatherings | **0.4753** | -0.0343 | 0.1316 |
| Participates in community meetings | **0.7297** | 0.0397 | 0.0294 |
| Participates in burial/funeral | **0.8506** | -0.0292 | -0.0343 |
| **Physical health** |  |  |  |
| I always have difficulties in hearing | 0.1236 | **0.4291** | -0.0430 |
| I always have difficulties in remembering | 0.1476 | **0.4609** | 0.0710 |
| I always have difficulties in moving on my own | -0.0241 | **0.5978** | -0.0362 |
| I always have difficulties with feeding myself | 0.0296 | 0.3876 | -0.4025 |
| I always have difficulties with taking a bath or shower | -0.1358 | **0.4256** | -0.2604 |
| I always have difficulties while dressing | -0.0066 | **0.4525** | -0.3821 |
| I have been with body pain in the past one month | -0.0563 | **0.4010** | 0.1880 |
| I have experienced pain around the joints in past 3 months | -0.1257 | **0.5322** | 0.1311 |
| I easily get tired even when I have not done much work | -0.1244 | **0.4869** | 0.1567 |
| I have enough energy for everyday life | 0.1662 | -0.4028 | -0.1104 |
| I always have difficulties in sleeping | 0.0100 | **0.5980** | 0.0331 |
| **Intimacy** |  |  |  |
| I have opportunities to love other people | 0.0880 | 0.0508 | **0.6141** |
| I am loved by other people around me | 0.0635 | 0.0682 | **0.7142** |
| I live happily with my family members | -0.0585 | -0.0062 | **0.6177** |
| I no longer engage in sexual relations | -0.2097 | 0.1561 | 0.1531 |
