# Supplementary figures and images for: Predictors of quality of life of older persons in rural Uganda: A cross sectional study
Source: AAS Open Res. 2018 Nov 9;1:22. Originally published 2018 Jul 26. [Version 2] doi: 10.12688/aasopenres.12874.2 (PMC7118782; doi:10.12688/aasopenres.12874.2)

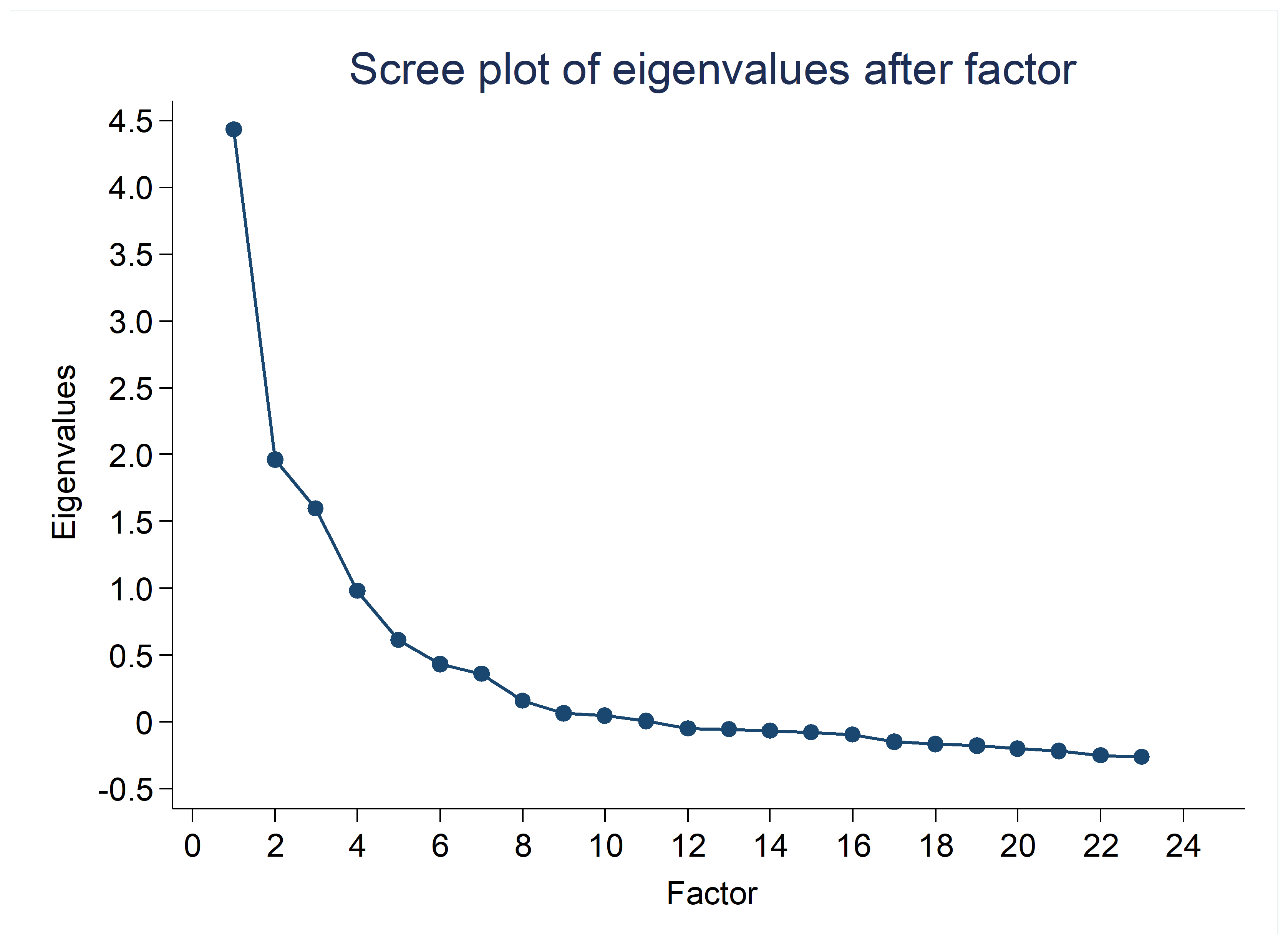

Supplement: Supplementary file 2 [file aasopenres-1-13995-s0001.tgz › 0a88fb04-e811-41f2-838e-d947e3d900e7.png]
