# Supplementary material for: Predictors of quality of life of older persons in rural Uganda:  A cross sectional study
Source: AAS Open Res. 2018 Nov 9;1:22. Originally published 2018 Jul 26. [Version 2] doi: 10.12688/aasopenres.12874.2 (PMC7118782; doi:10.12688/aasopenres.12874.2)
Supplement: Peer review history

# Peer review: Reviewer response for version 2

**DOI:** 10.21956/aasopenres.13995.r26762

**Published:** 2019-02-19

**Authors:** Robert G Cumming

**Affiliations:** 1 School of Public Health, University of Sydney, Camperdown, NSW, Australia

This is a good piece of research on quality of life of older people in rural Uganda. However, I think it could be improved.

 A statistician needs to check the section concerned with Factor Analysis and the construction of the final 3-level quality of life variable. I think it is probably ok but, because it is fundamental to the validity of the paper, it should be reviewed by an expert.    

 Other issues:

1. There are too many abbreviations. What does SAGE stand for? No need to use "OP" for older people or "FA" for factor analysis. Better to spell out in full. 
2. In a cross-sectional study, it is better say "associated with" rather than "predict".
3. "Population ageing" means an increase in the proportion of the population above a certain age (usually 60). While it is true that population ageing is occurring rapidly in developing countries in Asia and the Americas, it is not true in sub-Saharan Africa. Yes, the absolute number of older people is increasing very rapidly in sub-Saharan Africa but, because the fertility rate is still very high, the proportion of older people will remain relatively low for many years into the future. 
4. What was the participation rate? How many people were excluded because they were too ill?
5. How many questions related to quality of life were in the final study questionnaire? The two WHO questionnaires have a total of 50 questions (26+24) but I assume that not all were used in this study.
6. To my knowledge, the WHOQOL instruments are usually used to construct a score between 0 and 100. Can the authors please comment on why they did not use this approach?
7. The sentence at the foot of column 1 on page 4 that begins "Western region .." is confusing. It reads as if Western region had the highest proportion with tertiary education, residing in semi-permanent homes (etc) but I think the authors mean that people with tertiary education/residing in semi-permanent homes (etc) were more likely to have poor quality of life.
8. Some of the results in table 3 are surprising and merit discussion. For example, those living alone were much more likely to have good quality of life than those living with a spouse. It also seemed that the further you live from a health centre, the better the quality life. Those using firewood for fuel had better quality of life than those using charcoal. Also surprising that those with tertiary education had poorer quality of life.

I confirm that I have read this submission and believe that I have an appropriate level of expertise to confirm that it is of an acceptable scientific standard, however I have significant reservations, as outlined above.

# Peer review: Reviewer response for version 2

**DOI:** 10.21956/aasopenres.13995.r26637

**Published:** 2018-12-03

**Authors:** Rose Nathan

**Affiliations:** 1 Ifakara Health Institute (IHI), Dar es Salaam, Tanzania

The study evaluated factors that predict Quality of life of older persons in rural Uganda using cross-sectional survey data from four regions. Considering the epidemiologic and demographic transitions happening in most sub-Saharan African countries including the aging population; this subject is timely and useful for informing policies. Indeed studies have shown that perceived quality of health is a predictor or associated to subsequent mortality in adults ^1^.

 Introduction cited relevant literature on the subject and knowledge gap to be addressed by the current study is clearly stated. Aim of the study is clear.

 The study appropriately adopted World Health Organization Quality of Life (WHOQoL) tool to measure quality of life and suitable statistical methods were applied to determine predictors of quality of life.

 Results are clearly presented in a logical flow consistent with the analytical approach and the aim of the study.

 Discussion fairly subjected the results in the light of existing literature. However, it is rather surprising that age did not appear as a significant predictor of quality of life. A study across several countries (lower and middle income), age appeared consistently as a predictor of quality of life[ ^2^.

 Conclusion is reasonably drawn from the findings.

*A minor comment*

 I would propose to move the first sentence in the discussion to the end of the discussion (variables that did not show statistical significance as predictors of quality of life).

I confirm that I have read this submission and believe that I have an appropriate level of expertise to confirm that it is of an acceptable scientific standard.

## References

[1] : Is self-rated health an independent index for mortality among older people in Indonesia?. *PLoS One*.2012;7(4) : 10.1371/journal.pone.0035308 e35308 10.1371/journal.pone.0035308 22523584PMC3327655

[2] : Variations in disability and quality of life with age and sex between eight lower income and middle-income countries: data from the INDEPTH WHO-SAGE collaboration. *BMJ Glob Health*.2017;2(4) : 10.1136/bmjgh-2017-000508 e000508 10.1136/bmjgh-2017-000508 29333288PMC5759706

# Peer review: Reviewer response for version 1

**DOI:** 10.21956/aasopenres.13940.r26581

**Published:** 2018-09-04

**Authors:** Rose Nathan

**Affiliations:** 1 Ifakara Health Institute (IHI), Dar es Salaam, Tanzania

The study evaluated factors that predict Quality of life of older persons in rural Uganda using cross-sectional survey data from four regions. Data was collected using the WHO-QOL instrument with some modifications. Considering the epidemiological and demographic changes happening in most sub-Saharan African countries including the raising population of the aged, this subject is timely and useful for informing policies.

**Methods**: A more comprehensive description of the data weighting and description as to how data analysis accounted for the weighted data due the sampling scheme (multi-stage, cluster) is useful to convince audience that the results are credible.

   

**Results:** While results showed significantly higher odds of good quality of life for OPs residing in permanent houses, implying a better standard of living; other household parameters that are considered to reflect standard of living showed inverse relationship to quality of life among the OPs. Such indicators included electricity as source of energy, borehole/piped water as source of water and type of toilet (covered pit latrine). Need to revisit this or provide some explanation to these seemingly contradictory results.

 Add confidence intervals to the ORs.

**Discussion**: It is worth mentioning in the discussion variables that showed negative association with quality of life or failed to show a significant association contrary to empirical knowledge documented by several other studies.  On this list are: distance to health facility, age of the participant and marital status.  

**Limitation**: Exclusion of those who were ill could have potentially biased the results.

I confirm that I have read this submission and believe that I have an appropriate level of expertise to confirm that it is of an acceptable scientific standard, however I have significant reservations, as outlined above.

# Peer review: Reviewer response for version 1

**DOI:** 10.21956/aasopenres.13940.r26550

**Published:** 2018-08-23

**Authors:** Zarina Nahar Kabir

**Affiliations:** 1 Karolinska Institute, Stockholm, Sweden

The research focuses on an important group of population, the elderly population, who are largely ignored in research and policy making in low-income countries. The study investigated the factors associated with quality of life of older persons in the different regions of Uganda. In order to assess quality of life, the instrument developed by WHO has been used in a modified version. Some specific comments are given below:

 Introduction: It is stated that that older persons in Uganda experience various challenges including loneliness, poor housing and financial challenges but there are no relevant references to corroborate this statement.

 Methods: Those who were "ill" were excluded from the study. Two questions arise from this. Firstly, how was being "ill" defined? Secondly, it is highly likely that exclusion of this group will introduce bias in the results by affecting the outcome. If data is available on health or morbidity status of the sample, in addition to HIV/AIDS, this should be included in the analysis and the results presented. If the data is not available, it is important to discuss the consequences in the limitations of the study.

 It is not clear if the districts and villages were randomly selected.

 I advise the authors to present the 95% Confidence Interval for the Odds Ratios presented in Table 4.

 There is some repetition in the description of the dependent/outcome variable. This should be avoided.

 Conclusions and policy implications: The recommendation of interventions to focus on older persons' HIV/AIDS "pandemic" based on the limited information and weak association presented in the study is far-fetched. Researchers need to be cautious when suggesting interventions if the results may merit further investigation due to weak associations and/or ommission of important factors such as morbidity status in the current study.

 Language review: I suggest that the manuscript goes through a language review.

I confirm that I have read this submission and believe that I have an appropriate level of expertise to confirm that it is of an acceptable scientific standard, however I have significant reservations, as outlined above.
